# Quantitative Analysis of Lung Ultrasonography for the Detection of Community-Acquired Pneumonia: A Pilot Study

**DOI:** 10.1155/2015/868707

**Published:** 2015-02-25

**Authors:** Francesco Corradi, Claudia Brusasco, Alessandro Garlaschi, Francesco Paparo, Lorenzo Ball, Gregorio Santori, Paolo Pelosi, Fiorella Altomonte, Antonella Vezzani, Vito Brusasco

**Affiliations:** ^1^Anaesthesia and Intensive Care Unit, Galliera Hospital, 16100 Genoa, Italy; ^2^Department of Internal Medicine and Medical Specialties, University of Genoa, 16100 Genoa, Italy; ^3^Department of Surgical Sciences and Integrated Diagnostics, University of Genoa, 16100 Genoa, Italy; ^4^Radiology Department, IRCCS San Martino-IST, 16100 Genoa, Italy; ^5^Radiology Department, Galliera Hospital, 16100 Genoa, Italy; ^6^Department of Surgical Sciences and Integrated Diagnostics, IRCCS San Martino-IST, University of Genoa, 16100 Genoa, Italy; ^7^Department of Emergency Medicine, IRCCS San Martino-IST, 16100 Genoa, Italy; ^8^Department of Surgery, University Hospital of Parma, 43100 Parma, Italy

## Abstract

*Background and Objective*. Chest X-ray is recommended for routine use in patients with suspected pneumonia, but its use in emergency settings is limited. In this study, the diagnostic performance of a new method for quantitative analysis of lung ultrasonography was compared with bedside chest X-ray and visual lung ultrasonography for detection of community-acquired pneumonia, using thoracic computed tomography as a gold standard. *Methods*. Thirty-two spontaneously breathing patients with suspected community-acquired pneumonia, undergoing computed tomography examination, were consecutively enrolled. Each hemithorax was evaluated for the presence or absence of abnormalities by chest X-ray and quantitative or visual ultrasonography. *Results*. Quantitative ultrasonography showed higher sensitivity (93%), specificity (95%), and diagnostic accuracy (94%) than chest X-ray (64%, 80%, and 69%, resp.), visual ultrasonography (68%, 95%, and 77%, resp.), or their combination (77%, 75%, and 77%, resp.). *Conclusions*. Quantitative lung ultrasonography was considerably more accurate than either chest X-ray or visual ultrasonography in the diagnosis of community-acquired pneumonia and it may represent a useful first-line approach for confirmation of clinical diagnosis in emergency settings.

## 1. Introduction

Community-acquired pneumonia in adults is a common, potentially life-threatening disorder with high hospitalization rate [1, 2]. Moreover, it is the only acute infection of the respiratory tract where delayed antibiotic therapy has been associated with an increased risk of death [3]. Therefore, a timely diagnosis is mandatory. A recent study showed that in 22% of patients admitted to emergency departments with a clinical diagnosis of pneumonia there were diagnostic uncertainties eventually causing delay in antibiotic treatment [4].

Based on the latest European Respiratory Society Guidelines [5, 6], pneumonia is defined as an acute illness with signs or symptoms compatible with a respiratory tract infection supported by radiological evidence of lung infiltrates. There is a strong consensus that chest X-ray (CXR) should be performed in all patients admitted with suspected pneumonia [6] because medical history and physical examination cannot provide sufficient evidence [7]. In emergency settings, however, the use of CXR may have major limitations due to patient conditions, waste of time, and interobserver variability [8]. Therefore, in critically ill patients with suspected pneumonia, the use of computed tomography (CT) scan is recommended [9, 10]. But CT is not always easily available in all emergency departments and is limited by exposure risks and costs [11, 12].

Lung ultrasonography (LUS) has also been proposed for detection of pneumonia [13–17], but it is still not widely accepted in clinical practice [18, 19]. This is because LUS has been generally validated by comparison with CXR, which is recognized to have low specificity, whereas CT was only sporadically used as comparator. Although there is agreement that LUS is suitable for detecting consolidations directly or via related artifacts, the diagnosis of pneumonia may be missed in about 8% of cases [14, 17], possibly due to limitations of visual analysis. Indeed, reflection artifacts may be weak or even absent and thus not detectable by eye when consolidation is small or far from pleural line. Quantitative analysis of ultrasonography is an objective method that has been clinically applied to different organs but not yet to lungs [20]. In a previous recent study [21], quantitative lung ultrasonography (QLUS) proved to be an accurate method to evaluate extravascular lung water in a model of pulmonary edema. It can be hypothesized that QLUS may also be useful for detection of pulmonary consolidations of different origins.

The aim of this study was twofold: (1) to validate the diagnostic accuracy of QLUS in the diagnosis of community-acquired pneumonia by using non-contrast-enhanced CT as reference method and (2) to compare QLUS with semiquantitative LUS visual analysis and CXR.

## 2. Methods

### 2.1. Patients

During a 6-month study period (April–September 2012) 207 consecutive patients with suspected community-acquired pneumonia were admitted to the emergency department of IRCCS-Azienda Ospedaliera Universitaria-IST of Genoa. Pneumonia was clinically suspected on the basis of cough, dyspnea, body temperature >38°C or <35°C, heart rate >90 beats/min, tachypnea >20 breaths/min, rales or crackles on auscultation, and abnormal oxygen saturation [6, 22]. In all cases, hospitalization was required based on clinical criteria [23]. CURB-65 severity score was calculated and recorded at hospital admission for all patients included in the study [24]. Patients with pro-BNP positive were excluded from the study. Thirty-two patients (17 male, 15 female; mean age 62 ± 19 yr, range 21–96 yr) undergoing CXR, LUS, and CT studies and pneumonia diagnosis confirmed by at least one of them were included in the study. The indications for CT were suspected pulmonary embolism, discrepancy between clinical signs of pneumonia and negative CXR, or severity. The CXR criteria for diagnosis of pneumonia were the following: (1) homogeneous consolidation abutting the visceral pleura and with lobar or sublobar extension, (2) patchy or nodular pattern, and (3) streaky pattern [25]. The LUS criteria were the following: (1) presence, distribution, and extent of artifacts suggestive of interstitial involvement, (2) pleural line abnormalities, and (3) alveolar consolidation. The CT criteria were the presence of at least one of the following: (1) segmental or peribronchovascular scattered ground glass, (2) reticular opacities, and (3) consolidation compatible with acute-phase lung involvement [25].

The study was approved by the local ethics committee (number 3/2012) and all patients gave informed written consent to participate.

### 2.2. Measurements

All patients underwent a standard single-view anterior-to-posterior CXR and LUS within 1 h from admission. CXR was obtained in a supine or semirecumbent position using a mobile device (Dr 9000 System Kodak Direct View, Italy). LUS was performed by a Logiq-e unit (GE Healthcare, Milwaukee, WI) with broadband convex-array probe at 4 MHz and high-frequency linear-array probe at 10 MHz. Patients underwent LUS in a supine or semirecumbent position for anterior fields and seated or lateral position for posterior-lateral fields, depending on clinical conditions. Each hemithorax was scanned over every intercostal space along the conventional parasternal, midclavicular, axillary, and paravertebral lines [18]. The transverse scan was used to better visualize the pleural line, avoiding acoustic interference from the ribs. One LUS image for each intercostal space of all scanned areas was stored as uncompressed DICOM.

CT without contrast medium was obtained as soon as available, but not later than 1 h from LUS. CT scans were obtained by a GE Light Speed 16 slice (Fairfield, CT) set at 130 kVp, 200 mAs, 6 × 1.0 mm collimation, 1.50-pitch factor, and 50 cm data collection diameter. Reconstruction parameters were 5.0 mm slice thickness and medium smooth convolution kernel (B41s). Scanning was performed from apex to base with 0.8 s rotation time (pitch factor 0.5–1.8) and 16 mm feed/rotation. Images were stored as uncompressed DICOM files at standard 512 × 512 pixel resolution for quantitative analysis. Quantitative CT analysis was also performed using dedicated software (Maluna, Mannheim Lung Analyzing Tool, version 2.02, Mannheim, Germany) to determine volume of nonaerated lung parenchyma, after manual segmentation procedure.

**Figure 1:**
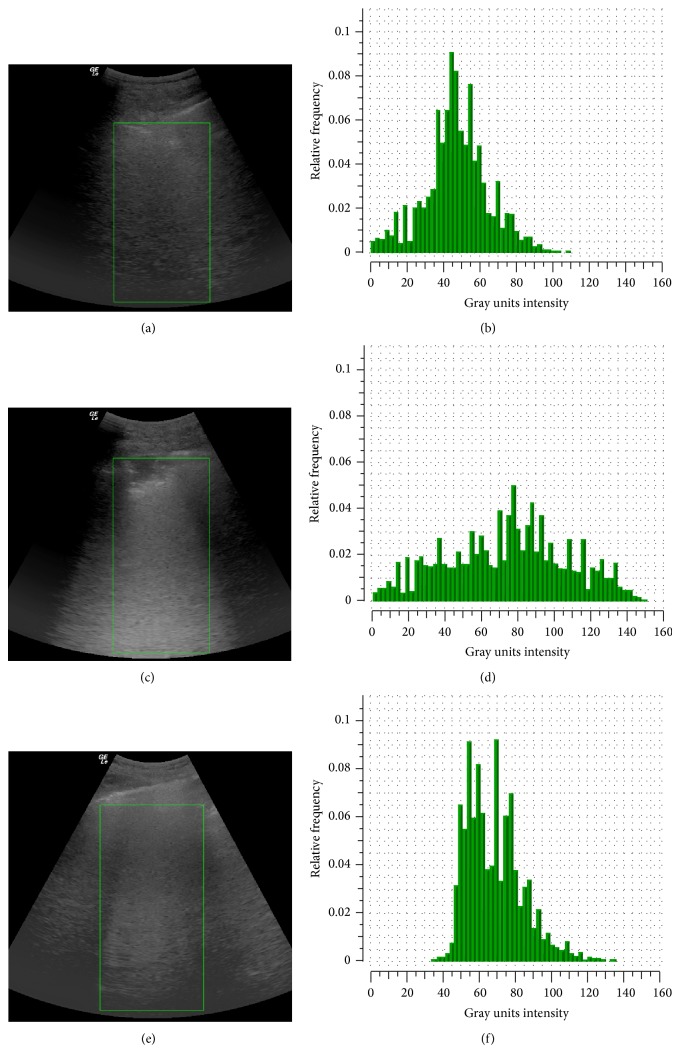
Representative echo images (left) and frequency distribution of Gray scale units by quantitative analysis (right) for three patients with normal hemithorax (upper panels), subpleural (middle panels), and non-subpleural (lower panels) consolidations.

QLUS analysis was performed using the single-frame image stored with the following settings: 52 DB gain, focus at 16 cm with convex-array probe to maximize ultrasound beam collimation, 50% time-gain compensation, 1-dynamic range, removal of 2nd harmonic, and automatic postprocessing to avoid artifact attenuation (cross × beam). QLUS was evaluated by a computer-assisted grayscale analysis (QUANTA Critical Care, CAMELOT Biomedical Systems Srl (http://www.quanta.camelotbio.com/)). A region of interest area was chosen extending from the pleural line to the bottom of the image (Figure 1) and the echo intensity was determined for each of the 0.2 mm horizontal slices down from the top. The frequency distribution of echo intensities (Gray units) for the whole image was then calculated and the mean value retained for subsequent data analysis.

For the purposes of the study, all images were reviewed to select those lung regions where at least one of the usual methods, that is, CXR or LUS or CT, showed signs of pneumonia. CT was the gold standard for true positive and true negative results.

### 2.3. Statistical Analysis

All variables were expressed as mean ± standard deviation (SD) or percentage (%). The normality of sample distribution was verified by applying Shapiro-Wilk and D'Agostino-Pearson omnibus test. Categorical data were compared using Pearson *χ* ^2^ test and Fisher's exact test. Continuous variables were compared with nonparametric Wilcoxon signed-rank test or Friedman test. The best cutoff value for QLUS was determined by ROC analysis assuming a binomial distribution [26, 27]. Sensitivity, specificity, positive (PPV) and negative (NPV) predictive values, and accuracy were calculated by standard formulas. Concordance between imaging methods was analyzed by Cohen test [28], considering the agreement to be fair if *k* values were from 0.21 to 0.40, moderate from 0.41 to 0.60, and substantial from 0.61 to 0.80 [29]. Correlation between mean echo intensity (Gray units) and nonaerated lung parenchyma determined by quantitative CT was evaluated with Spearman rank test. A logistic regression analysis was performed to identify potential predictors for mean echo intensity by retaining only the significant model that passed the goodness-of-fit test [30]. Statistical significance was assumed with two-tailed *P* values < .05. Statistical analysis was carried out using SPSS version 20.0 (SPSS Inc., Chicago, IL) and the R software/environment (R Foundation for Statistical Computing, Vienna, Austria); at the time of this paper, R-3.0.2 was available.

## 3. Results

Based on CT findings, 14 of the 32 patients included in the study had bilateral pneumonia. All of them were spontaneously breathing with a CURB-65 severity score of 1.7 ± 1.2 (range 0–4). Of the 64 hemithoraxes examined, 44 showed alveolar consolidations and 20 no signs of pneumonia at CT scan. CXR identified 32 pneumonia, LUS 31, and QLUS 40. In 5 CT-negative hemithoraxes, CXR was falsely positive in 4, LUS in 1, and QLUS in 1 (Table 1). In the remaining 15 CT-negative cases, CXR, LUS, and QLUS were consistently negative. Sensitivity was of 64% for CXR, 68% for LUS, and 77% for their combination (Table 2).

**Figure 2:**
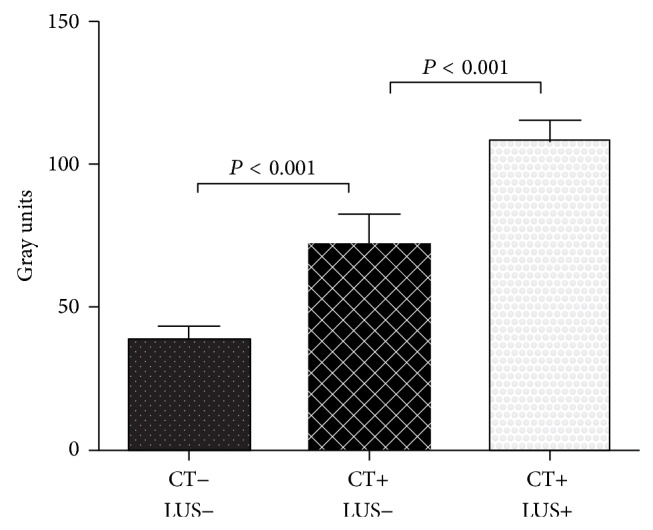
Mean echo intensity (Gray units) by quantitative lung ultrasonography from nonaffected (CT−) and affected (CT+) hemithoraxes with positive (LUS+) or negative (LUS−) results. CT: computed tomography; LUS: visual lung ultrasonography.

QLUS provided mean values of Gray units significantly lower in CT-negative (39 ± 9) than CT-positive (93 ± 26) hemithoraxes (*P* < .001). When all CT-positive hemithoraxes were divided into two subgroups based on LUS results, the mean Gray unit (Figure 2) was significantly (*P* < .001) higher in the LUS-positive (103 ± 21) than LUS-negative (73 ± 22) ones. Both subgroups were significantly different from the CT-negative group (*P* < .001). The best Gray Unit cut-off determined by ROC analysis (AUROC .971, SE .020, *P* < .001, and 95% CI: .932–1.000) was 48, returning 95% sensitivity and 90% specificity. By using this cut-off for diagnosis of pneumonia, the sensitivity of QLUS was of 93% (Table 2).

There were differences in results depending on site of pneumonia, with those of lower lobes being identified less often by CXR and LUS (*P* < .01) and those of upper lobes rarely assessed by LUS (*P* < .05). By contrast, pneumonia localized at middle lobes was identified almost equally by all the three methods. QLUS did not show differences between upper and lower or ventral and dorsal lung areas (*P* > .99) (Table 3).

As compared with CXR-negative pneumonias, the CXR-positive pneumonias were significantly larger in size (84 ± 56 versus 28 ± 21 mm; *P* < .01) and closer to the pleural line (3 ± 8 versus 22 ± 20 mm; *P* < .001), independent of site. As compared with LUS-negative pneumonias, the LUS-positive pneumonias were significantly larger in diameter (81 ± 55 versus 28 ± 26 mm; *P* < .001) and closer to the pleural line (1 ± 3 versus 28 ± 23 mm; *P* < .001). In two cases LUS missed a subpleural pneumonia localized in the upper anterior lobe.

QLUS yielded 3 falsely negative and 1 falsely positive results but detected 15 pneumonias not identified by LUS. Mean Gray units were significantly correlated with size of the consolidation (*r* = .63, *P* < .001), volume of nonaerated lung calculated by quantitative CT (*r* = .79, *P* < .001), and distance from pleural line (*r* = −.77, *P* < .001). After including these variables as independent predictors in univariate logistic regression models for pneumonia by QLUS, the statistical significance was reached only for volume of nonaerated lung calculated by quantitative CT (*β* = .079; odds ratio = 1.08 (95% CI from 1.03 to 1.14); *P* = .004) and distance from pleural line (*β* = −.0552; odds ratio = .95 (95% CI from .9 to .99); *P* = .028). However, of these two independent variables only the nonaerated lung pass the goodness-of-fit test and was therefore retained in the model.

## 4. Discussion

The main findings of this study are the following: (1) CXR and LUS, either separately or combined, showed low accuracy in the diagnosis of pneumonia; (2) the accuracy of CXR was limited by location and size of the consolidation; (3) the accuracy of LUS was limited by distance from pleural line, location, and size of consolidation; (4) by contrast the use of QLUS increased the accuracy in detecting pneumonia independent of size and distance from pleura.

To our knowledge, this is the first study describing a novel quantitative and objective method to analyze lung ultrasonography in humans. The accuracy of QLUS was 94%, much greater than CXR or LUS or their combination. This was probably because QLUS is less influenced by the distance from pleura. In 14 hemithoraxes with non-subpleural pneumonia, mean echo intensity was higher than in healthy hemithoraxes. Presumably, this may reflect an increased number of air-to-fluid interfaces formed by neutrophil-rich exudate within small partially aerated zones surrounding the consolidation area, yet insufficient to increase CT physical density and too far from pleura to generate B-lines. Mean echo intensity was also higher than normal where pneumonia was even detected by LUS, presumably because of the coexistence of consolidated parenchyma and perilesion edema resulting in a more hyperechogenic image. QLUS was strongly correlated with the quantity of nonaerated parenchyma determined by CT quantitative analysis. In two cases (numbers 4 and 28) QLUS intensity was similar despite different distance from pleura and size of consolidation. An explanation for these findings might be that a smaller lesion may have the same echogenicity as a larger one because of different underlying pathology, for example, alveolar versus interstitial. Moreover, quantitative CT reflects the average density of pneumonia and surrounding lung parenchyma, which may vary depending on preexisting regional ventilation-to-perfusion ratio.

In the present study, the sensitivity of LUS in identifying parenchymal consolidation was much lower than previously reported (59 versus 88–95%) [19, 31, 32]. A likely explanation for this difference may be the selection criteria used in the present study, whereby only patients requiring CT scan were included (32 of 207, 15%). Indications for CT included discrepancy between clinical diagnosis and negative or inconclusive CXR. By contrast, in previous studies [13–17], the clinical diagnosis of pneumonia was confirmed by CXR and CT was used in a minority of cases when LUS and CXR were discordant. In these studies, the percentage of pneumonia not reaching the pleura was 6 to 8%, which is less than what is found in the present study (17 out of 44) and previously reported by using CT [11]. It can be therefore speculated that the low performance of LUS in the present study is due to the inability to detect non-subpleural pneumonias detected by CT. In a previous study of critically ill patients [33], LUS had a very high accuracy in detecting consolidations due to different causes, which was explained by the fact that most of these reached the pleura. Moreover, in critically ill ventilated patients lying supine for several days, consolidations are more likely to occur in posterior-basal lung regions and thus are easier to be detected by LUS. Therefore these findings cannot be directly extrapolated to spontaneously breathing patients with suspected community-acquired pneumonia, as those of the present study. In fact the present study shows that the accuracy of LUS depends on distance from pleura and size of consolidation, confirming a very high accuracy only in the case of consolidations <4 mm from pleural line. The interposition of aerated parenchyma between pleural line and consolidation makes visualizing even large consolidated areas or typical LUS artifacts impossible.

The diagnostic accuracy of either CXR or LUS is limited by anatomic structures such as diaphragm, liver, heart, and vertebrae causing image superimposition. In the present study, CXR more frequently missed consolidations of lower than upper or middle lobes, whereas the opposite was the case for LUS. This is likely because CXR lung images may be confounded by diaphragm, liver, heart, and vertebral images, whereas shoulder blades, supraclavicular fossa, and axillary region are difficult to explore by LUS due to probe positioning.

Moreover, CXR assessment may be problematic in supine or semirecumbent position because of the difficulty to obtain full lung inflation and a lateral view. Indeed, in anterior-posterior CXR images with patients lying supine or semirecumbent, as usually obtained in emergency settings, the dome of the diaphragm projects itself over a significant portion of anterior and basilar lung fields [34].

Although CT is traditionally considered as the gold standard in the evaluation of lung consolidations, it is indicated only in a limited number of patients admitted to hospital for suspected pneumonia, that is, in those with severe signs and symptoms of pneumonia, suspicion of severe complications, worsening of symptoms, discrepancy between imaging and clinical findings, or particularly extensive consolidations at CXR [5, 6, 12]. Major problems connected with CT are radiation exposure, costs, and need to move the patient to radiology department. Although the effective radiation dose of the spiral chest CT is nowadays 3.5 mSv, that is, 70 times that of anterior-posterior CXR [35], and new reduced radiation dose CT protocols might reduce the radiation-exposure problem [36], there are still concerns regarding the safety of repeated chest CT scanning and this is one reason why IDSA/ATS Guidelines on the management of community-acquired pneumonia still do not recommend the use of CT [6, 37].

This study has some limitations. First, the study was conducted on a small number of cases of pneumonia (15% of patients admitted to the emergency department with clinical diagnosis of pneumonia) because patients performed CT scan only for clinical reason. Second, CXR, LUS, and QLUS comparisons were performed “a posteriori” on the single frames corresponding to topographical areas where pneumonia was detected by CT. This was a choice in the design of the study, which was intended as a proof of concept aimed at describing the ability of QLUS in detecting pneumonia independent of size and distance from pleura on those areas with definite pneumonia. The analysis was performed retrospectively for two reasons: the software of recent construction was allocated in a remote PC and provides the analysis of DICOM images previously stored. This allowed analyzing LUS and CXR obtained in a double-blind fashion, as it occurs in clinical practice. Therefore, further studies in unselected patients are required to confirm the clinical usefulness of real-time QLUS over the whole lung surface. Third, the mean GU values obtained in the present study using a single device cannot be generalized to other ultrasound devices with different settings, beam profiles, and focuses. Therefore, future developments of QLUS, such as second-order texture-analysis, are advocated and will be helpful to further increase its accuracy independent of machine settings.

In conclusion, the results of this pilot study introducing QLUS for detection of community-acquired pneumonia are encouraging as this technique allowed recognizing 41 out of 44 pneumonias diagnosed by CT. It cannot be expected that QLUS will replace CT as the gold standard for the diagnosis of pneumonia because it does not provide a precise estimate of the size of consolidation and a whole assessment of both lungs. Nevertheless, QLUS might become a suitable method for confirming clinical diagnosis and for bedside monitoring of patients with community-acquired pneumonia as a useful complement to visual LUS with a significant increase of diagnostic accuracy.

## Figures and Tables

**Table 1:** Imaging data in patients with community-acquired pneumonia.

Patient number	Lung region	CT	CXR	LUS	QLUS	Distance from pleura^*^	Lesion size^*^	Nonaerated volume^*^	Gray units^#^
						(mm)	(mm)	(mL)	
1	RLL	+	+	+	+	0	85	237	90

2	RML	+	+	+	+	0	55	150	115
	Lingula	+	+	+	+	0	100	163	118

3	RLL	+	−	+	+	0	17	70	105
	RLL	+	+	+	+	0	75	235	106

4	RLL	+	−	−	+	90	11	40	73
	LUL	+	−	−	−	30	10	29	41

5	LLL	+	−	−	+	20	22	69	72
	RLL	+	+	+	+	0	27	93	112

6	RLL	+	−	−	+	22	52	243	60
	LLL	+	−	+	+	0	20	210	80

7	RML	+	+	+	+	0	150	300	150

8	RLL	+	+	+	+	11	58	110	60

9	RUL	+	+	−	−	37	35	32	48

10	LLL	+	−	−	+	13	18	73	73
	RUL	+	+	−	+	18	44	137	83

11	RML	+	−	+	+	0	52	750	123
	LUL	+	+	+	+	0	125	826	114

12	LLL	+	−	+	+	0	66	180	93

13	LLL	+	−	−	+	15	10	270	108
	RLL	+	+	+	+	5	61	235	93

14	RLL	+	−	−	−	60	8	32	39

15	LUL	+	+	−	+	0	107	368	109
	RLL	+	−	+	+	0	79	128	109

16	LW	+	+	+	+	0	195	839	129
	RLL	+	+	+	+	0	93	180	107

17	RLL	+	+	+	+	11	42	89	77

18	RLL	+	+	+	+	0	37	157	92

19	RLL	+	+	+	+	10	37	75	58

20	RW	+	+	+	+	0	200	640	123
	LW	+	+	+	+	0	230	720	142

21	RLL	+	+	+	+	0	56	90	95
	LLL	+	+	+	+	0	26	142	113

22	RLL	+	+	+	+	0	131	1074	100
	LLL	−	+	−	−	—	—	—	47

23	RLL	−	−	+	−	—	—	0	30

24	LLL	−	+	−	−	—	—	0	47

25	LUL	+	+	+	+	0	34	115	97

26	LLL	+	+	+	+	0	69	300	87

27	RUL	+	+	−	+	0	11	189	87
	LW	−	+	−	−	—	—	0	46

28	RW	+	+	+	+	0	110	469	121
	LLL	+	−	−	+	26	20	260	71

29	RLL	+	−	+	+	0	28	178	80
	LLL	+	+	+	+	0	91	200	106

30	LLL	+	−	−	+	40	14	73	71
	RLL	+	−	−	+	7	32	107	80

31	RML	+	+	+	+	0	71	210	94

32	RLL	−	+	−	+	—	—	0	52

CT: spiral computed tomography; CXR: chest X-ray; LUS: lung ultrasonography; QLUS: quantitative LUS; ^*^determined by CT; ^#^determined by QLUS. RW: whole right lung; LW: whole left lung; RUL: right upper lobe; RML: right middle lobe; RLL: right lower lobe; LUL: left upper lobe; LLL: left lower lobe.

**Table 2:** Diagnostic accuracy of imaging methods for community-acquired pneumonia.

	CT+	CT−	*S*	Sp	PPV	NPV	DA	*k*
CXR+	28	4	.64	.80	.88	.50	.69	.38
CXR−	16	6						

LUS+	30	1	.68	.95	.97	.58	.77	.54
LUS−	14	19						

CXR or LUS+	34	5	.77	.75	.85	.75	.77	.49
CXR or LUS−	10	15						

QLUS+	41	1	.93	.95	.98	.86	.94	.85
QLUS−	3	19						

*S*: sensitivity; Sp: specificity; PPV: positive predictive value; NPV: negative predictive value; DA: diagnostic accuracy; *k*: Cohen *k* test.

Other abbreviations are as in Table 1.

**Table 3:** Number of positive findings in relation to localization.

Lung regions	Number of structures			
	CT	CXR	LUS	QLUS
Right side				
Upper lobe	5	5	2	5
Middle lobe	6	5	6	6
Lower lobe	19	12	15	18

Left side				
Upper lobe	5	4	3	4
Lingula	4	4	4	4
Lower lobe	12	6	8	12
Paracardiac	1	0	0	1

Abbreviations are as in Table 1.
